# Peroxisome Proliferator-Activated Receptor γ Activity is Required for Appropriate Cardiomyocyte Differentiation 

**DOI:** 10.22074/cellj.2016.4317

**Published:** 2016-05-30

**Authors:** Maryam Peymani, Kamran Ghaedi, Shiva Irani, Mohammad Hossein Nasr-Esfahani

**Affiliations:** 1 Department of Biology, Science and Research Branch, Islamic Azad University, Tehran, Iran; 2 Department of Biology, Faculty of Sciences, University of Isfahan, Isfahan, Iran; 3 Department of Cellular Biotechnology, Cell Science Research Center, Royan Institute for Biotechnology, ACECR, Isfahan, Iran

**Keywords:** Embryonic Stem Cell, PPARγ, Differentiation

## Abstract

**Objective:**

Peroxisome proliferator-activated receptor γ (PPARγ) is a member of the PPAR nuclear receptor superfamily. Although PPARγ acts as a master transcription factor in adipocyte differentiation, it is also associated with a variety of cell functions including carbohydrate and lipid metabolism, glucose homeostasis, cell proliferation and cell differentiation. This study aimed to assess the expression level of *PPARγ* in order to address its role in cardiac cell differentiation of mouse embryonic stem cells (mESCs).

**Materials and Methods:**

In this an intervening study, mESCs were subjected to cardiac differentiation. Total RNA was extracted from the cells and quantitative real time polymerase chain reaction (qPCR) was carried out to estimate level of gene expression. Furthermore, the requirement of PPARγ in cardiac differentiation of mESCs, during cardiac progenitor cells (CPCs) formation, was examined by applying the respective agonist and antagonist.

**Results:**

The obtained data revealed an elevation in the expression level of *PPARγ* during spontaneous formation of CPCs and cardiomyocytes. Our results indicated that during CPC formation, PPARγ inactivation via treatment with GW9662 (GW) reduced expression of CPC and cardiac markers.

**Conclusion:**

We conclude that PPARγ modulation has an effective role on cardiac differentiation of mESCs at the early stage of cardiomyogenesis.

## Introduction

Peroxisome proliferator-activated receptors (PPARs) are ligand-activated transcription factors belonging to the superfamily of transcription nuclear receptors. They consist of three major isoforms, termed α, β/δ and γ, encoded by distinct single-copy genes. Previous studies have well documented the function of PPARs as regulators of lipid and lipoprotein metabolism, and glucose homeostasis (1). Moreover, they influence numerous cellular events including proliferation, differentiation and apoptosis (1,2). The importance of PPARs in cardiac function and development has been shown by many studies in recent years (2,5). PPARα, which is highly expressed in high energy demand tissues such as heart, has a close association with energy turnover. During the process of cardiac development and under physiological/pathophysiological stimuli, PPARα regulates genes encoding mitochondrial fatty acid β-oxidation (FAO) enzymes (6). On the other hand, down-regulation of PPARα markedly reduces expression of cardiac sarcomeric proteins and specific genes, thus preventing cardiac differentiation (7). Therefore, treatment with PPARα agonists (WY14643, GW7647 and ciprofibrate) significantly enhances cardiomyogenesis and increases expression of cardiac genes. Furthermore, ubiquitously expressed PPARβ/δ can exert anti-inflammatory and anti-hypertrophic effects in cardiomyocytes (6). Despite the low expression of PPARγ in the heart, it may be essential for normal cardiac development as its loss from the heart or its activation by an external agonist results in cardiac hypertrophy (2). In addition, activation of PPARs may play a critical role during cardiomyogenesis of embryonic stem cells (ESCs) as they express *PPAR* transcripts during cardiac differentiation (6). 

Cardiomyocyte differentiation from ESCs is a complicated process under regulation of various cardiac specific transcription factors and signaling molecules which occurs into two stages, namely cardiogenesis and cardiac myofibrillogenesis (6). Several studies have shown that cardiac progenitor cells (CPCs) have a potency to differentiate into all three major cell types of the heart: cardiac myocytes, smooth muscle cells and endothelial cells (8,10). During cardiomyocyte differentiation of mouse ESCs (mESCs), the cardiac transcription factors (*Gata4, Nkx2.5* and *Mef2C*) start to be fully expressed. Then, the cardio precursor cells emerge regardless of presence of those genes encoding the sarcomeric proteins (α-MHC, MLC2V). However, at the end of the cardiomyocyte differentiation process, both cell type markers are identifiable (11). 

mESCs are well recognized as pluripotent stem cell lines with an ability to differentiate into all three primary germ layers of ectoderm, mesoderm and endoderm during development (12,13). The advancement of technology and the ability of culturing mESCs *in vitro* have made them an appropriate cell line for transplantation therapy, molecular genetic, drug discovery and developmental studies (13). Development of cardiac cells from mESCs is well characterized to be under the regulation of signaling molecules and cardiac specific transcription factors which include zinc-finger GATA proteins and Nkx-2.5 (6,7). 

Although a prominent role has been proposed for PPARγ during cardiac cell development, there is limited knowledge about the significance of *PPARγ* expression in cardiac differentiation of mESCs. The present study aimed to investigate the significance of activation/inactivation stages of PPARγ in cardiac cell differentiation by using mESCs as a model system. 

## Materials and Methods

This study is was an intervening investigation approved by the Ethics Committee of Royan Institute. Chemicals were supplied by Life Technologies (USA) unless indicated otherwise. 

### Culture method and spontaneous differentiation of mouse embryonic stem cells to beating

cardiomyocytes mESCs, Royan B1 cell lines derived from the C57BL/6 strain (14), were kept in an undifferentiated state in Knock-out Dulbecco’s Modified Eagle Medium (Ko-DMEM) supplemented with 15% ESC-qualified fetal calf serum (ES-FCS) as previously reported (15,16). The differentiation of cardiac cells was induced through the hanging drops method in order to form embryoid bodies (EBs) for two consecutive days as previously described (16). After EBs collection, they were placed in suspension culture for the next five days (16,17). On the seventh day, the collected EBs were placed on gelatin coated 12-well plates (Techno Plastic Products, Switzerland). For better results, the cultures were kept in the presence of neurobasal medium, supplemented with 15% ES-FCS, 0.1 mM non-essential amino acids, 2 mM L-glutamine, 0.1 mM beta-mercaptoethanol, 1% penicillin-streptomycin and B-27 supplement for the next 8 days. 

### Embryoid body treatment by PPARγ agonist and antagonist

Rosiglitazone (Rosi, Cayman Chemical, USA) was used as a potent specific PPARγ agonist (18,19) and GW9662 (GW, Sigma-Aldrich, USA) (20,22) as a specific antagonist. Both were dissolved in dimethyl-sulfoxide (DMSO). An equal amount of solvent was used in all samples including the control group. Involvement of PPARγ on the early and late stages of cardiac differentiation was evaluated through treating EBs with the effective concentrations of Rosi (5 µM) and GW (10 µM) (17) respectively. This evaluation was carried out during the five day period of the suspension culture and the week of post plating in Ko-DMEM (supplemented with 15% ES-FCS medium) respectively. 

### Quantitative real-time polymerase chain reaction analysis

Total RNA of the cells was extracted using RNeasy Mini Kit (Qiagen, Germany). To generate cDNA, Moloney murine leukemia virus (MMLV) reverse transcriptase, 1 µg of each RNA sample and random hexamer primers were used according to the manufacturer’s protocol. Using SYBR Green (TaKaRa, Japan), qPCR was carried out in a Rotor gene 6000 thermal cycler (Corbett, Australia) as suggested by the protocol. The PCR mixture contained 10 µl RotorGene SYBR Green PCR Master Mix, 3 pmole of each primer, and 25 ng of cDNA for each reaction in a final volume of 20 µl. The relative mRNA concentrations were calculated using the software provided by the manufacturer and normalized to *β-tubulin* V expression level. All measurements were done in triplicate and expression level changes were reported according to the ΔΔCt method. Specific primer pairs (Table 1) were designed by the Beacon designer (Version 7.2, USA) and Perl-primer, and ordered through Metabion company (Martinsried, Germany). 

### Immunocytochemistry analysis

An indirect immunofluorescence light microscopy was used to analyze the cells as previously described (16,17). The primary antibodies were antimouse antibodies against α-Cardiac actin (1:800, Sigma-Aldrich, USA). The secondary antibodies were Tetramethyl-rhodamine-isothiocyanate (TRITC)-conjugated goat anti-mouse IgG (1:50, Chemicon, USA). In parallel, the nuclei were counterstained with 4´,6-diamidino-2-phenylindole (DAPI, Sigma-Aldrich, USA). The stained cells were analyzed with a fluorescent microscope (Olympus, Japan) and images were acquired with an Olympus DP70 camera (Olympus, Japan). 

### Characterization of embryoid bodies

The number of beating EBs was measured in each dish in three different experiments. Furthermore, the area ratio of beating EBs was measured by taking the mean size of 10 separate beating EBs in each group respective to controls (Table 2). 

**Table 1:** Primers used for gene expression analysis by quantitative real-time polymerase chain reaction


Genes	Sequencing primer (5´-3´)	Annealing temp (˚C)	Accession no.

Mesp1	F:CAGTCCCTCATCTCCGCTCT	62	NM_008588.2
	R: CAGTCCCTCATCTCCGCTCT		
Nkx2.5	F:TTAGGAGAAGGGCGATGAC	57	NM_008700.2
	R: AGGGTGGGTGTGAAATCTG		
Mef2c	F:CGAGTGTAAGTGTCTAATG	54	NM_001170537.1
	R: CCTATTGTCAGAATTGCTAT		
α-Cardiac actin	F:GTGTGACGACGAGGAGAC	61	NM_009608.3
	R: GTGTGACGACGAGGAGAC		
α-MHC	F:CAGAAGCCTCGCAATGTC	58	NM_001164171.1
	R: CGGTATCAGCAGAAGCATAG		
SMAα	F:TCAGGGAGTAATGGTTGGAATG	61	NM_007392.2
	R: TTGGTGATGATGCCGTGTTC		
*SM22α*	F:TGTCACTCCTGTTAGCATTCC	54	NM_011526.5
	R: GGTCACTCTTCTTCTCCATAGC		
Gapdh	F:TGCCGCCTGGAGAAACC	58	NM_008084.2
	R: TGAAGTCGCAGGAGACAACC		
PPARγ	F:TGAGACCAACAGCCTGAC	60	NM_001127330.1
	R: GTTCACCGCTTCTTTCAAATC		


**Table 2:** Characterization of embryoid bodies (EBs)


GW 10 µM	Rosi 5 µM	Control	Criteria

Percentage of beating EBs	80 ± 4	70 ± 3	15 ± 6
Relative area of beating EBs compare to the control	100	120 ± 13	60 ± 21


### Statistical analysis

SPSS (version 17) was used to express data as means ± SEM obtained from three independent treatments of the replicated observations. One-way ANOVA was applied to assess globally statistical difference among multiple treatments. Also independent t test analysis was carried out to identify statistical differences between any two treatments. P<0.05 (*) was taken as the level of statistical significance. 

## Results

### Expression analysis of PPARγ during spontaneous cardiomyocyte differentiation

**Fig.1:**
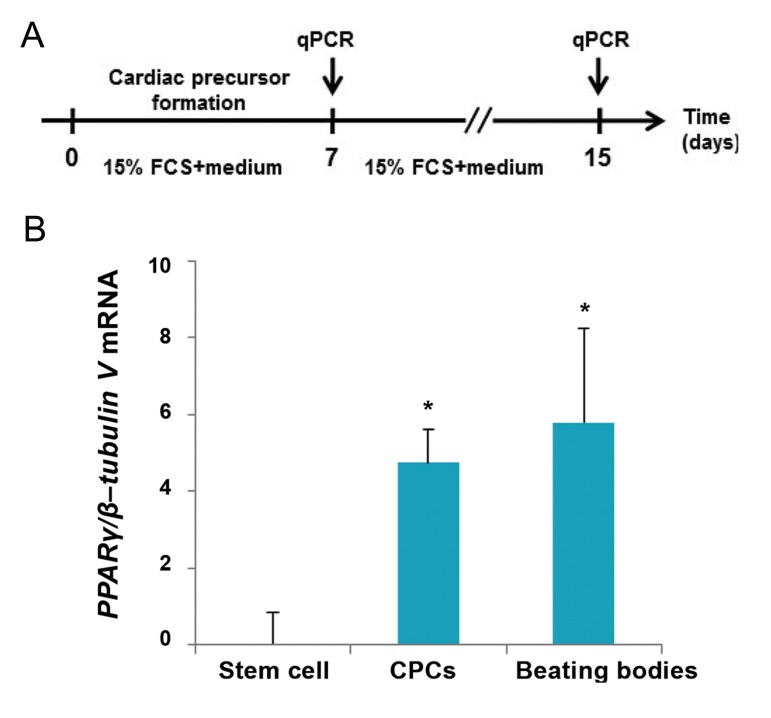
Increased level of peroxisome proliferator-activated receptor γ (*PPARγ*) expression during spontaneous cardiac differentiation of mouse embryonic stem cells (mESCs). A. Illustrated protocol of mESCs during cardiac differentiation: early embryoid bodies (EBs, day 2) were cultured in fetal calf serum (FCS) enriched media for additional 5 days. Emerged cardiac progenitor cells (CPCs) were allowed to differentiate within 8 days and B. Quantitative real-time polymerase chain reaction (qPCR) analysis of PPARγ expression level of mESCs (stem cell), CPCs (day 7) and beating bodies (day 15). Relative expression level of PPARγ was quantified and normalized with β-tubulin V. Represented value bars are the mean of triplicate independent experiments ± SEM. *; Indicates a significant difference between the treated and control groups at day 7 and day 15 (P<0.05).

To access *PPARγ* expression level during spontaneous cardiomyocyte differentiation (Fig.1A), qPCR was performed in three different steps within 15 days on mESCs (stem cell: day 0), CPCs (day 7) and cardiomyocytes (day 15). Results showed an increasing trend of *PPARγ* expression level which reached its highest level on day 15 (Fig.1B). 

### Effects of PPARγ agonist and antagonist treatment during cardiac progenitor cells formation

**Fig.2:**
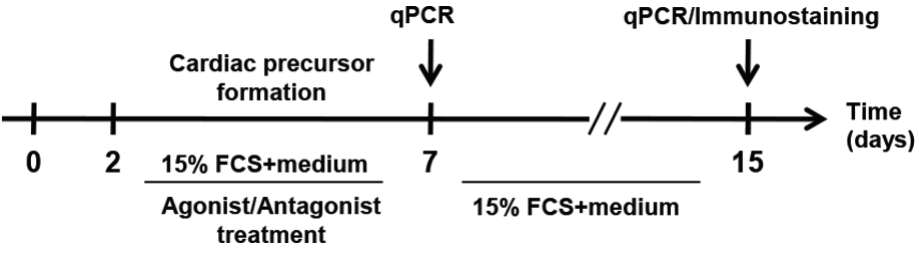
llustrated protocol of mouse embryonic stem cells (mESCs) treatment with peroxisome proliferator-activated receptor γ (*PPARγ*) agonist [Rosiglitazone (Rosi)] and antagonist [GW9662 (GW)] during cardiac progenitor cells (CPCs) formation (within the five day interval). qPCR; Quantitative real-time polymerase chain reaction and FCS; Fetal calf serum.

**Fig.3:**
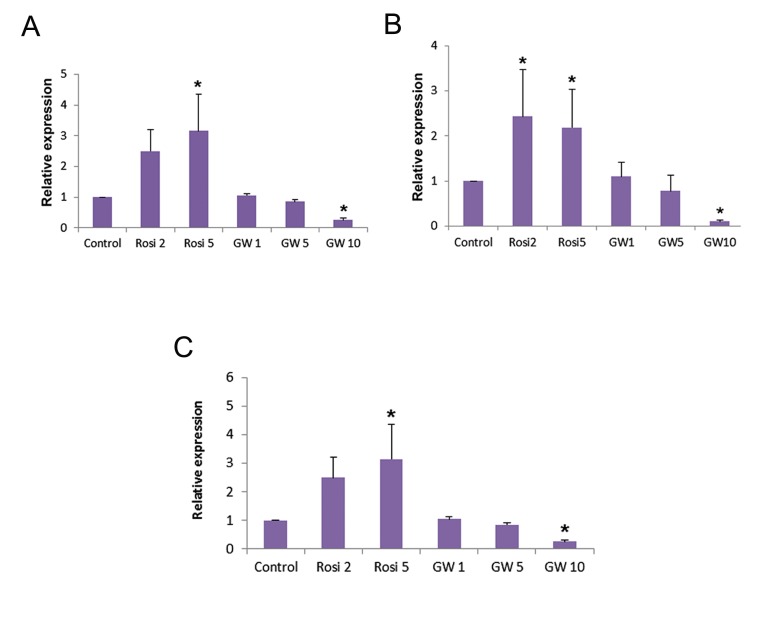
Modulation of peroxisome proliferator-activated receptor γ (PPARγ) activity during cardiac progenitor cell (CPC) formation. Quantitative real-time polymerase chain reaction (qPCR) analysis of A. *Tbx5 , B. Nkx2.5,* and *C. Mef2c* (CPC marker) expression level on day seven of the experiment. Relative expression level of these genes was quantified and normalized with β-tubulin V. *; Indicates a significant difference between the treated and control groups (P<0.05).

 To our knowledge, cytosolic PPARγ is translocated to the nucleus once activated (23,24). The effectiveness of agonist (5 µM Rosi) and antagonist (10 µM GW) concentrations for PPARγ activation and inactivation respectively was already confirmed. Thus, cardiac precursor cells were treated with the aforementioned components as previously described (17). To investigate the correlation between *PPARγ* expression level and its activation/inactivation, mESCs were treated with different concentrations of the respective agonist and antagonist during CPC formation (Fig.2). Both concentrations (2 and 5 µM) of Rosi caused a significant increase in the expression level of *Nkx2.5, Tbx5* and *Mef2c* (CPCs markers) on day 7 [(Fig.3), Rosi 2 and 5 µM]. Moreover, 10 µM of GW [(Fig.3), GW10 µM)] decreased the transcript level of this marker significantly when compared with the control (untreated) group. 

**Fig.4:**
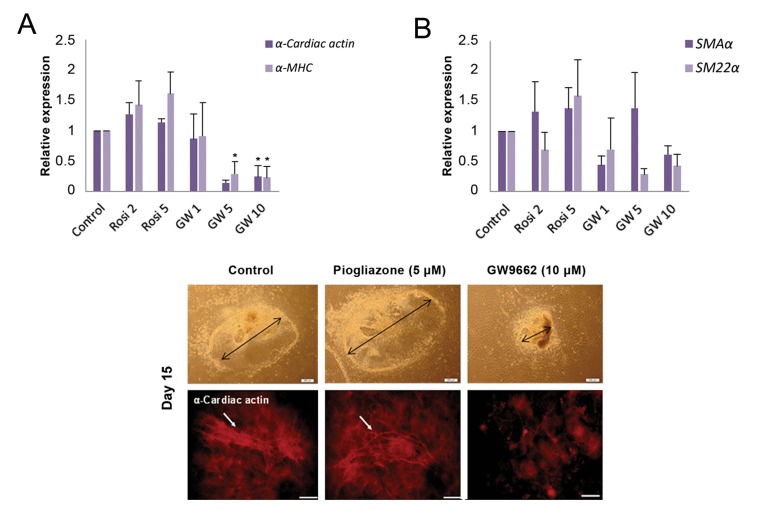
Modulation of peroxisome proliferator-activated receptor γ (PPARγ) activity on cardiac cell and smooth muscle cell markers. A. Quantitative real-time polymerase chain reaction (qPCR) analysis of *α-Cardiac actin* and *α-MHC* (cardiac cell markers) in the beating bodies, B. qPCR quantitative analysis of *SMAα* and *SM22α* (smooth muscle cell markers) in the beating bodies. The relative expression level of target genes were quantified and normalized with β-tubulin V. *; Indicates a significant difference between the treated and control groups (P<0.05), C-E. Morphological illustration of the generated beating bodies. The reduced size of beating bodies was observed in the GW9662 (GW)-treated samples and F-H. Immunostaining of the generated beating bodies derived from the treated cardiac precursor cells and the control group against α-Cardiac actin on day 15 of the experiment (scale bar; 200 μm).

The differentiation procedure used in this study was a sort of bi-potential differentiation type. To evaluate the effect of PPARγ on the differentiation rate of myocardial and smooth muscle cells, expression level analysis of cardiac markers *α-cardiac actin* and *α-MHC*, and smooth muscle cell markers like *SM22α* and *SMAα* were evaluated on day 15. In the agonisttreated cells, the expression of cardiomyocyte markers (*α-cardiac actin* and *α-MHC*) were not significantly affected compared with the control, whereas the antagonist, at concentrations of 5 and 10 µM, caused a sharp decrease in the expression of the aforementioned markers (Fig.4A). Interestingly, both PPARγ agonist and antagonist treatments did not have any significant effects on the expression of smooth muscle markers (*SM22α* and *SMAα*) (Fig.4B). This indicates that the differentiation rate of myocardial cells but not smooth muscle cells was decreased upon inactivation of PPARγ during CPC formation. 

Agonist and antagonist treated CPCs were allowed to differentiate to cardiac cells as described above (Fig.2). On day 15, spontaneous cardiomyocyte differentiation was assessed based on the morphological features of the beating cells. As shown in Figure 4C-E, the expansion size of the beating cardiac cells in the Rosi-treated sample was comparable to that found in the control sample but dissimilar to cells in the GW-treated sample. Moreover, immunostaining of the beating cardiac cells with an antibody against α-cardiac actin (a cardiomyocyte marker) confirmed the data obtained by morphological analysis (Fig.4F-H). 

## Discussion

PPARγ activation/inactivation and its potential impact on cardiomyopathic remodeling and cardiophysiology have been intensively reviewed (25). PPARγ deficiency in cardiomyocytes resulted in severely impaired cardiac structure which leads to cardiac dysfunction mainly associated with the elevated oxidative stress-induced mitochondrial abnormalities (7) whereas dependency of appropriate cardiac function to PPARγ could be confronted by overexpression of *PPARγ* (26). 

This study was carried out to address the involvement of PPARγ in different stages of cardiac differentiation. We recently established the stagedependent role of PPARγ modulation on neural differentiation of mESCs by retinoic acid treatment (17). In here, we gained further insight into the expression of *PPARγ* during the spontaneous cardiomyocyte differentiation of mESCs in two stages of cardiac and post-CPC formation. Despite the relatively low expression of *PPARγ* in the heart, it exerts a prominent role in cardiac function (24,27). Our data revealed that *PPARγ* expression increased during CPC formation and remained at a high level even at the terminal stage of cardiac differentiation. This result is in concordance with the data reported by Sharifpanah et al. (6) whose results showed maximal expression of *PPARγ* on days 8 and 18. We then divided the spontaneous cardiomyocyte differentiation in two stages of CPC and cardiomyocyte formation. As depicted on day 7, CPCs were formed as evident by an increase in the expression level of the cardiac transcription factor *Nkx2.5.* Furthermore, on day 15, the highest level of *PPARγ* expression level was detected in differentiated cardiomyocytes. To investigate whether this increased level of PPARγ plays a role in cardiyomyogenesis, respective agonist and antagonist treatments were carried out during CPC formation. Our data displayed that the agonist significantly induced the activation of PPARγ which resulted in overexpression of *CPC* marker *Nkx2.5* on day 7 while the respective antagonist caused a reverse effect. Our results are in contradiction with those of Sharifpanah et al. (6) who showed that PPARγ agonists have no effect on cardiomyogenesis. This inconsistency may be due to different procedures for cardiomyogenesis and different strategies in undertaking expression analysis. However, further experiments are required to clarify this issue. 

## Conclusion

We conclude that PPARγ influences spontaneous cardiomyocyte differentiation from mESCs at the early stages when CPCs are forming. This conclusion could be an asset along with that of a previous study which indicated that pioglitazoneactivation of PPARγ was followed by improvement of cardiomygenic transdifferentiation of human mesenchymal stem cells. Taken together, PPARγ influences the potentiation of cell commitment at the early stages of cardiomyogenesis. However, further research is required to investigate how PPARγ inactivation inhibits heart cell differentiation. 
